# PHD finger proteins function in plant development and abiotic stress responses: an overview

**DOI:** 10.3389/fpls.2023.1297607

**Published:** 2023-11-17

**Authors:** Wenli Quan, Zhulong Chan, Piwei Wei, Yahui Mao, Dorothea Bartels, Xun Liu

**Affiliations:** ^1^ College of Bioengineering, Sichuan University of Science and Engineering, Yibin, China; ^2^ Institute of Molecular Physiology and Biotechnology of Plants (IMBIO), University of Bonn, Bonn, Germany; ^3^ Key Laboratory of Horticultural Plant Biology, Ministry of Education, Key Laboratory of Urban Agriculture in Central China, Ministry of Agriculture, College of Horticulture and Forestry Sciences, Huazhong Agricultural University, Wuhan, China; ^4^ College of Life Science and Technology, Hubei Engineering University, Xiaogan, China

**Keywords:** PHD finger, methylation, transcription regulator, plant development, abiotic stress, molecular mechanism

## Abstract

The plant homeodomain (PHD) finger with a conserved Cys4-His-Cys3 motif is a common zinc-binding domain, which is widely present in all eukaryotic genomes. The PHD finger is the “reader” domain of methylation marks in histone H3 and plays a role in the regulation of gene expression patterns. Numerous proteins containing the PHD finger have been found in plants. In this review, we summarize the functional studies on PHD finger proteins in plant growth and development and responses to abiotic stresses in recent years. Some PHD finger proteins, such as VIN3, VILs, and Ehd3, are involved in the regulation of flowering time, while some PHD finger proteins participate in the pollen development, for example, MS, TIP3, and MMD1. Furthermore, other PHD finger proteins regulate the plant tolerance to abiotic stresses, including Alfin1, ALs, and AtSIZ1. Research suggests that PHD finger proteins, as an essential transcription regulator family, play critical roles in various plant biological processes, which is helpful in understanding the molecular mechanisms of novel PHD finger proteins to perform specific function.

## Introduction

The plant homeodomain (PHD) finger is a common zinc-binding domain that existed in all eukaryotic genomes (Bienz, 2006). The PHD finger is usually comprised of 50–80 amino acids and typically has a conserved Cys4-His-Cys3 motif containing insertion sequences with various length and composition in the domain (Aasland et al., 1995; Bienz, 2006). The PHD finger exhibits high sequence similarity to the RING finger (Cys3-His-Cys4 type), which also binds to two zinc atoms (Capili et al., 2001). Previously, some RING fingers were mistaken for PHD fingers, leading to the incorrect conclusion that PHD fingers were also involved in E3 ligase activity (Wei et al., 2015). In general, the three-dimensional structure of the PHD finger shows a globular fold, consisting of an alpha-helix and a two-stranded beta-sheet.

Histone acetyltransferases (HATs) catalyze histone acetylation and play an important role in the positive epigenetic regulation of gene expression in eukaryotes (Zhang et al., 2023). Ever since *Arabidopsis* HAT3.1 was identified as the first PHD finger protein (Schindler et al., 1993), many PHD finger proteins have been found in fungus, animals, and plants (Martin et al., 2006; Baker et al., 2008; Hu et al., 2018). Most of these proteins are localized in the nucleus (Aasland et al., 1995; Gilbert et al., 2014; Shu et al., 2015), while a few are predicted to be localized in the membrane, including the chloroplast thylakoid membrane (Glyma10g05080.1) and mitochondrial inner membrane (Glyma11g11720.1) (Wu and Wang, 2014). A typical PHD finger protein usually contains one or more PHD finger domains. As an independent structure unit, most PHD finger-containing proteins only have PHD finger domain (Wu and Wang, 2014). However, there are other conserved domains in a certain protein concomitant with PHD finger domain, such as DUF3594 (Domain of Unknown Function 3594), BAH (Bromo Adjacent Homology), and DDT (DNA binding homeobox and different transcription factors) domain (Sun et al., 2017). These various domains individually cooperate with PHD finger domain to play a role in a particular biological event (Wu and Wang, 2014). The Alfin1 group belongs to a plant-specific subfamily of PHD finger proteins. Alfin1 from alfalfa (*Medicago sativa*) is a salt-induced transcription factor and can efficiently bind to the G-rich elements (GNGGTG/GTGGNG) in the promoter region of *MsPRP2*, a stress-responsive gene (Bastola et al., 1998). Overexpression of *Alfin1* in alfalfa increased the transcript of *MsPRP2* in roots and enhanced the tolerance of transgenic plants to salt stress (Winicov et al., 2004). So far, an increasing number of Alfin1-like (AL) proteins have been identified and characterized in various plant species, such as *Arabidopsis thaliana* (Wei et al., 2015), *Brassica rapa* (Kayum et al., 2015), and *Atriplex hortensis* (Tao et al., 2018). Except the conserved PHD finger domain in C-terminal, all AL proteins contain the conserved DUF3594 domain in N-terminal with unknown function. AL proteins containing the DUF3594 domain have not been found in animals, fungi, or prokaryotes (Tao et al., 2018).

PHD finger domains are proved to be involved in protein–DNA and protein–protein interaction (Bastola et al., 1998; Wei et al., 2017). The N-terminal tails of nucleosome core histones (H2A, HAB, H3, and H4) are usually modified by methylation or acetylation, which is called post-translational modification in histone proteins (PTM) (Lee et al., 2010; Yang et al., 2013; Sun et al., 2014). The PHD finger is a methyllysine and methylarginine “reader” domain, which can specifically recognize and bind to methylation marks in histone H3 (Sanchez and Zhou, 2011; Milosevich and Hof, 2016; Miura et al., 2020). Lee et al. (2009) first identified that PHD fingers in ING (inhibitor of growth) homologues AtING and AL proteins are able to bind histone H3 at di- or tri-methylated lysine4 (H3K4me2/me3) in *Arabidopsis*. Additionally, many PHD finger proteins are proved to be involved in chromatin remodeling and have transcriptional regulation activity (Bienz, 2006; Wei et al., 2015; Milosevich and Hof, 2016; Diego-Martin et al., 2022). Chromatin remodeling processes function in the control of gene expression patterns that modulate development in eukaryotic organisms (López-González et al., 2014). Thus, these “reader” proteins are essential for recruiting chromatin remodeling complexes and transcription factors to target loci and regulating their transcriptional status (López-González et al., 2014; Milosevich and Hof, 2016). In this way, the PHD finger proteins play important roles in translating histone modifications into downstream gene expression patterns.

PHD finger proteins function in various biological processes because of high sequence diversity except the eight conserved cysteine/histidine residues (Wei et al., 2015). In plants, diverse functions of PHD finger proteins have been characterized, which are involved in different biological processes, including the regulation of seed dormancy and germination (Ye et al., 2016), vernalization response (Sung and Amasino, 2004; Kim and Sung, 2013), flowering time (Qian et al., 2021), and pollen development (Yang et al., 2019a; Yang et al., 2019b). Furthermore, many genes encoding PHD finger proteins can be induced by environmental stresses and participate in plant abiotic stress responses (Wei et al., 2009; Gao et al., 2018; Alam et al., 2019; Pang et al., 2022). In this review, we aim to summarize and analyze the functions of PHD finger proteins in plants, particularly in plant reproduction development and responses to abiotic stresses. This will provide useful information for studying novel PHD finger proteins and further exploring the molecular mechanisms of these proteins involved in specific biological events.

## Roles of PHD finger proteins in plant growth and development

The processes of plant growth and development play vital roles in plant reproduction and the completion of its life cycle. Previous studies showed that lots of PHD finger proteins are involved in these biological events, such as the regulation of flowering time, pollen development, seed germination, metabolite biosynthesis, and metal transport (**Table 1**).

**Table 1:** PHD finger proteins involved in plant growth and development.

PHD finger protein	Plant species	Domain	Involved in plant growth and development	Reference
VIN3	*Arabidopsis thaliana*	PHD finger, FNIII domain, VID	Flowering time	Sung and Amasino, 2004
VIL1	*Arabidopsis thaliana*	PHD finger, FNIII domain, VID	Flowering time	Sung et al., 2006
AtSIZ1	*Arabidopsis thaliana*	SAP, PHD finger, PINIT, SP-RING, SXS	Floral repressor	Jin et al., 2008
SHL	*Arabidopsis thaliana*	PHD finger, BAH domain	Flowering time	Müssig et al., 2000; López-González et al., 2014
EBS	*Arabidopsis thaliana*	PHD finger, BAH domain	Flowering time	López-González et al., 2014
PFP	*Arabidopsis thaliana*	PHD type zinc finger, UBR type zinc finger	Flowering time	Yokoyama et al., 2019
OsVIL1, 2	*Oryza sativa* (rice)	PHD finger, FNIII domain, VID	Flowering time	Yang et al., 2013; Jeong et al., 2016
OsTrx1	*Oryza sativa* (rice)	PHD finger, SET domain	Flowering time	Choi et al., 2014
Ehd3	*Oryza sativa* (rice)	PHD finger	Flowering time	Matsubara et al., 2011
CaVIL1	*Capsicum* spp. (pepper)	PHD finger, FNIII domain, VID	Flowering time	Mohan et al., 2018
AIPP2/PAIPP2	*Arabidopsis thaliana*	N-terminus, PHD-PBR, C-terminus	Flowering time	Zhang et al., 2020
AtMS1	*Arabidopsis thaliana*	PHD finger, Leu zipper-like motif	Tapetum development and pollen wall formation	Wilson et al., 2001
HvMS1	*Hordeum vulgare* (barley)	PHD finger, Leu zipper-like motif	Tapetum development and pollen wall formation	Gómez and Wilson, 2014
CA05g06780 (MS1)	*Capsicum annuum* (paprika)	PHD finger, Leu zipper-like motif	Tapetum development and pollen wall formation	Jeong et al., 2018
ZmMS7	*Zea mays* (maize)	PHD finger, Leu zipper-like motif	Tapetum development and pollen wall formation	Zhang et al., 2018
OsPTC1/OsMS1	*Oryza sativa* (rice)	PHD finger, Leu zipper-like motif	Tapetum development and pollen wall formation	Li et al., 2011; Yang et al., 2019a
TIP3	*Oryza sativa* (rice)	PHD finger	Tapetum development and pollen wall formation	Yang et al., 2019b
MS3	*Glycine max* (soybean)	PHD finger	development of male gametophytes	Hou et al., 2022
BrMS1	*Brassica rapa* L. ssp. *Pekinensis* (Chinese cabbage)	PHD finger	Tapetum development and pollen wall formation	Dong et al., 2022
DUET/MMD1	*Arabidopsis thaliana*	PHD finger	Male meiosis	Andreuzza et al., 2015; Wang et al., 2016; 2020
Glyma.02G243200 (MS4)	*Glycine max* (soybean)	PHD finger	Male meiosis	Thu et al., 2019
AtAL6	*Arabidopsis thaliana*	DUF3596, PHD finger	Promote seed germination	Molitor et al., 2014
GSR1	*Arabidopsis thaliana*	PHD finger	Inhibit seed germination	Ye et al., 2016
PbPHD10	*Pyrus bretschneideri* (Chinese pear)	PHD finger, SNF, SANT	Lignin synthesis	Cao et al., 2018
MePHD1	*Manihot esculenta* Crantz (cassava)	PHD finger, BAH domain	Starch synthesis	Ma et al., 2018
OsTTA	*Oryza sativa* (rice)	PHD finger	Metal transport	Tanaka et al., 2018

### Flowering time

In plant species, the timing of the floral transition is a key developmental switch for the successful propagation. Flowering time is complexly controlled by genetic networks, epigenetic modifications, and other regulatory mechanisms (Khan et al., 2014; Sun et al., 2014). There exist different genetic pathways involved in the induction of flowering in *Arabidopsis*, such as the vernalizaiton pathway, the photoperiod pathway, and the gibberellin pathway (Khan et al., 2014). The expression of floral integrator genes, including *FLOWERING LOCUS T* (*FT*) and *SUPPRESSOR OF OVEREXPRESSION OF CO1* (*SOC1*), is finely controlled by these floral promoting pathways and floral repressors and then triggers floral initiation under proper conditions (Jarillo and Piñeiro, 2011).

As a floral repressor, *FLOWERING LOCUS C* (*FLC*) partly prevents flowering by repressing the expression of floral integrators in the first growing season for biennials and winter-annuals, while vernalization is necessary to promote flowering primarily by repressing *FLC* expression in the second growing season (Michaels, 2009). In *Arabidopsis*, VERNALIZATION INSENSITIVE 3 (VIN3) is a chromatin remodeling PHD finger protein and is required to repress *FLC* by promoting histone H3 deacetylation and increasing H3K9 and H3K27 methylation during vernalization (Sung and Amasino, 2004). VIN3-LIKE (VIL) proteins belong to *VIN3* gene family, containing the PHD finger domain, the fibronectin type-III (FNIII) domain, and the VIN3-interacting domain (VID). The PHD finger recognizes histone H3, while VID is responsible for the interaction between VIL proteins (Sung et al., 2006; Jeong et al., 2016). *AtVIL1-4* and *TmVIL1-3* genes are identified in *Arabidopsis* and wheat (*Triticum monococcum*), respectively (Fu et al., 2007). These VIL proteins play a crucial role in the flowering process regulated by vernalization and photoperiod pathways. For example, AtVIL1 cooperates with VIN3 in the chromatin modifications of *FLC* and *FLOWERING LOCUS M* (*FLM*, an *FLC*-related floral repressor) during vernalization. Prolonged cold treatment induces *VIN3* expression; however, the expression of *VIL1* is temperature independent and is highly upregulated in short days (SD). Indeed, *VIL1* promotes flowering in SD through the *VIL1*-mediated repression of *FLM* independent of *VIN3*. Thus, VIL1 involves in both the vernalization and photoperiod pathways by regulating expression of two floral repressors *FLC* and *FLM* (Sung et al., 2006). Wheat *VIL* genes are upregulated by vernalization and also affected by photoperiod (Fu et al., 2007). However, *CaVIL1* is an ortholog of *Arabidopsis VIL1* and functions as a flowering promoter in pepper (*Capsicum* spp.), which is insensitive to vernalization and photoperiod (Mohan et al., 2018). Vernalization is not required for flowering induction in rice (*Oryza sativa*), which contains four *VIL* genes (Fu et al., 2007). Among them, OsVIL2 physically associates with EMBRYONIC FLOWER 2b (OsEMF2b), which is a component of Polycomb Repressive Complex 2 (PRC2) with histone methyltransferase (HMTase) activity. The complex of OsVIL2-OsEMF2 induces flowering through epigenetic silencing of the flowering repressor *LEAFY COTYLEDON 2 and FUSCA 3-LIKE 1* (*OsLFL1*) with enriched H3K27me3 under both SD and long days (LD) (Yang et al., 2013). In addition, OsVIL2 interacts with OsVIL1, which is also associated with OsEMF2b to form a PRC2-like complex. Overexpressing of *OsVIL1* promotes flowering by reducing the transcripts of the flowering repressor *OsLF*, a bHLH transcription factor under SD, while it delays flowering by increasing expression of the flowering repressor *Grain number, plant height, and heading date 7* (*Ghd7*) under LD (Jeong et al., 2016).

In addition to VIL gene family, two *Arabidopsis* paralogs *SHORT LIFE* (*SHL*) and *EARLY BOLTING IN SHORT DAYS* (*EBS*), belonging to plant-specific transcriptional regulators with a PHD finger domain, function independently in the control of genes modulating flowering. PHD domains presented in SHL and EBS as chromatin effectors recognize H3K4me2/3 and bind to regulatory regions of the floral integrator genes *SOC1* and *FT*, respectively. Moreover, SHL and EBS are necessary to maintain the chromatin of *SOC1* and *FT* in an inactive conformation with low levels of H3 acetylation. These PHD finger proteins are proved to bind HISTONE DEACETYLASE 6 (HDA6) and play important roles in the chromatin-mediated repression of flowering, ensuring the precise control of flowering time (Müssig et al., 2000; López-González et al., 2014). Zhang et al. (2020) reported that the anti-silencing 1 (ASI1)-IMMUNOPRECIPITATED PROTEIN 2 (AIPP2) and PARALOG OF AIPP2 (PAIPP2) could independently interact with BAH domain-containing protein AIPP3 and PolII terminal domain (CTD) phosphatase (CLP2), respectively, through their PHD domain and C-terminus, to form the BAH-PHD-CLP2 (BPC) protein complex. The BPC complex combines the recognition of H3K27me3 and the repression of PolII release to repress the expression of *FT* in *Arabidopsis* to delay flowering (Zhang et al., 2020). Furthermore, it was confirmed that six PHD finger proteins in *Arabidopsis* can enhance the binding of BAH domain-containing transcriptional regulator 1 (BDT1) to the H3K27me3, which is essential for the prevention of early-flowering phenotype (Qian et al., 2021). An *Arabidopsis* PHD finger protein homolog, PFP (PHD finger domain containing protein), is critical for the flowering repression by upregulating expression of *FLC* and downregulating *FT* (Yokoyama et al., 2019). Some proteins only have the PHD finger domain, such as Early heading date 3 (Ehd3) in rice with encoding a nuclear protein containing two PHD finger motifs. As an LD preferential regulator, Ehd3 acts as a repressor upstream of *Ghd7* and promotes flowering under LD (Matsubara et al., 2011). It has been reported that Ehd3 associates with Trithorax 1 (OsTrx1), which carries a PHD finger motif and a SET domain with HMTase activity. The suppression of *OsTrx1* increases the transcripts of *Ghd7* and delays flowering time only under LD conditions (Choi et al., 2014).

### Pollen development

Male gametogenesis has important commercial significance for controlling the fertility of crops (Wilson et al., 2001). Microspore mother cells form tetrads after meiosis, and microspores with single nucleus are released from the tetrad. After nuclear fission, the microspores produce mature pollen grains. Acting as the innermost somatic cell layer of the anther locule, the tapetum plays a key role in pollen development (Ito and Shinozaki, 2002). *Arabidopsis* MALE STERILITY1 (MS1) functions as a transcriptional activator containing Leu zipper-like and PHD finger motifs, which are required for its function (Ito et al., 2007). The *MS1* gene is specifically expressed in the sporophytic tapetum for a short time and regulates the development of pollen exine and pollen cytosol and tapetum. The *ms1* mutant is male sterile and produces immature pollen with abnormal exine and tapetum (Wilson et al., 2001; Ito and Shinozaki, 2002; Ito et al., 2007).

Based on the information from pollen regulatory gene networks in *Arabidopsis*, several orthologs of *AtMS1* have been identified and functionally characterized in various species, such as *PERSISTENT TAPETAL CELL1*/*OsMS1* (*OsPTC1*/*OsMS1*) in rice (Li et al., 2011; Yang et al., 2019a), *HvMS1* in barley (*Hordeum vulgare*) (Gómez and Wilson, 2014), *ZmMS7* in maize (*Zea mays*) (Zhang et al., 2018), and *BrMS1* in Chinese cabbage (*Brassica rapa* L. ssp. *pekinensis*) (Dong et al., 2022). Using MutMap combined with KASP analysis, Dong et al. (2022) screened out a homologous gene of *AtMS1*, *BrMS1*, which plays a transcriptional regulatory role in tapetal programmed cell death (PCD) and pollen wall development. *ZmMS7*, encoding a PHD finger transcription factor in maize, shows 80.9% and 40.5% amino acid sequence identities with *OsPTC1*/*OsMS1* and *AtMS1*, respectively (Zhang et al., 2018). Mutation or overexpression of a barley ortholog of *AtMS1*, *HvMS1* results in male sterility. Under control of the native *AtMS1* promoter, *HvMS1* cDNA successfully complements the *Arabidopsis ms1* mutant, which demonstrates the conservation of *MS1* function in higher plants (Gómez and Wilson, 2014). Compared to the *Arabidopsis ms1* mutant, uncontrolled tapetal proliferation and subsequent necrosis-like tapetal death are uniquely displayed in the rice *ptc1* mutant (a single nucleotide insertion in the second exon of *LOC_Os09g27620*) (Li et al., 2011). Another research reported that the rice *osms1* mutant (four nucleotide deletion in the first exon of *LOC_Os09g27620*) shows significantly reduced transcripts of the genes related to tapetal PCD and pollen wall biosynthesis, including *AP25*, *AP37*, *EAT1*, *OsC4*, and *OsC6*. OsMS1 interacts with TDR INTERACTING PROTEIN2 (TIP2), a basic helix–loop–helix (bHLH) transcription factor, and OsMADS15, which are essential for sexual reproduction, through the PHD finger domain to regulate the tapetal PCD and pollen wall formation in rice (Yang et al., 2019a). Subsequently, Yang et al. (2019b) found that *TDR INTERACTING PROTEIN3* (*TIP3*) in rice encodes a PHD finger protein with the transcriptional activation activity. During another development, with the preferential accumulation in tapetum and microspores, TIP3 protein directly interacts with TDR, a bHLH transcription factor, which plays critical roles in the regulation of tapetum development and pollen wall formation. The loss of *TIP3* alters the transcript level of genes involved in tapetal PCD, biosynthesis, and transport of sporopollenin precursors, resulting in delayed tapetum degradation and no pollen wall formation in *tip3* mutant (Yang et al., 2019b).

Meiosis plays an important role in sexual reproduction, which produces haploid daughter cells essential for maintaining hybrid traits. This process involves two meiotic cell divisions, meiosis I and meiosis II, and each of both is divided into four stages, namely, prophase, metaphase, anaphase, and telophase. During meiosis, a complex series of biological events take place, including chromosome condensation, homologous chromosome recombination and segregation, and sister chromatid separation. The successful completion of meiotic events is necessary to form normal gametes. In *Arabidopsis*, *DUET* is also known as *MALE MEIOCYTE DEATH1* (*MMD1*), which encodes a nuclear protein containing a PHD finger and plays important roles in male meiosis. *DUET/MMD1* is specifically expressed in male meiocytes, coinciding with the time of meiosis (Reddy et al., 2003; Yang et al., 2003). Yang et al. (2003) showed that the *mmd1* mutant displays chromosome fragmentation in meiosis resulting in cell death of male meiocytes. Meanwhile, Reddy et al. (2003) indicated that the loss of *DUET* negatively affects chromosome condensation and male meiotic progression, leading to the formation of abnormal meiotic products. It was showed that DEUT/MMD1 binds to H3K4me2 *in vitro* and/or *in vivo* through the PHD finger domain, which is important for its functions in male meiosis (Andreuzza et al., 2015; Wang et al., 2016). Acting as a transcriptional regulator, DUET is specifically required for the expression of the meiotic gene *JASON* (*JAS*) and *THREE DIVISION MUTANT 1* (*TDM1*) critical for spindle organization during meiosis II and cell cycle exit after the second meiosis, respectively. Therefore, DUET functions in the regulation of microtubule organization and cell cycle transitions (Andreuzza et al., 2015). Recently, Liu et al. (2021) found that an *Arabidopsis* mutant *male meiotic restitution 1* (*mmr1*) is produced by an amino acid change G618D in the PHD finer domain caused by base conversion (C to T) at the third exon of *MMD1/DUET* gene. The hypomorphic mutant is deficient in spindle organization and mini-phragmoplast formation (Liu et al., 2021).

In addition, MMD1 is necessary to regulate the progression of chromosome condensation during meiotic prophase I. MMD1 PHD finger might directly bind to H3K4me2/3 at the *CAP-D3* promoter region to active the expression of *CAP-D3*, which is a condensin subunit gene belonging to the condensin complex required for chromosome condensation (Wang et al., 2016). Meanwhile, the MMD domain (a conserved domain in plants) of MMD1 interacts with the C-terminal FYR domain of Jumonji C (JmjC)-containing demethylase JMJ16 to broaden the substrate specificity of JMJ16 by binding the H3K9me3 in male meiocytes. Therefore, the interaction between MMD1 and JMJ16 demethylates H3K9 of target genes, for example, *CAP-D3*, and promotes gene expression, facilitating meiotic chromosome condensation (Wang et al., 2020). In soybean (*Glycine max*), *Glyma.02G243200* is isolated from one male-sterile, female-fertile mutant line (*ms4*) and named as MS4 protein, which is a homolog of *AtMMD1*. The *Arabidopsis mmd1* mutant with the soybean *MS4* gene restores successful tetrad formation and normal stamens and produces fertile pollen and viable seeds (Thu et al., 2019).

### Seed germination

Seed germination is a key developmental event that involves the timely transition from stagnant seeds to growing seedlings, which is vital for entering the plant life cycle. During this process, the expression of seed developmental genes needs to be gradually suppressed, such as *ABSCISIC ACID INSENSITIVE 3* (*ABI3*), *DELAY OF GERMINATION 1* (*DOG1*), and *CRUCIFERIN 3* (*CRU3*), to facilitate seedling growth in *Arabidopsis*. ABI3 is a plant-specific B3 domain transcription factor that regulates the expression of genes involved in seed development. CRU3 is one of 12S seed storage proteins, and its encoding gene is the direct target of ABI3. DOG1 plays a vital role in seed dormancy, and its expression in transcription and protein levels is strictly regulated during seed development (Molitor et al., 2014). The study showed that AtALs directly bind H3K4me3 regions at *ABI3* and *DOG1* loci through the PHD finger domain and also physically interact with the Polycomb Repressive Complex 1 (PRC1) RING-finger proteins AtRING1a and AtBMI1b through the N-terminal region. AL PHD-PRC1 complexes subsequently recruit PRC2 to establish H3K27me3 accumulation, resulting in a timely conversion from the H3K4me3-marked activation to the H3K27me3-marked repression of seed developmental genes during seed germination (Molitor et al., 2014). Germostatin (GS), a synthetic small molecule, is identified by chemical genetics approaches and can strongly inhibit seed germination through inducing auxin biosynthesis and enhancing auxin responses. GERMOSTATIN RESISTANCE LOCUS 1 (GSR1) with four tandem PHD finger domains binds to non-methylated H3K4 marks and is responsible for GS-induced prevention of seed germination. It physically interacts with AUXIN RESPONSE FACTOR 10/16 (ARF10/16) and IAA17 to form a co-repressor complex involved in auxin-mediated seed germination (Ye et al., 2016).

### Metabolite biosynthesis regulation

In cassava (*Manihot esculenta* Crantz), *ADP-glucose pyrophosphorylase small subunit 1a (MeAGPS1a*) is a significant catalytic subunit of ADP-glucose pyrophosphorylase (AGPase), which is the first enzyme in starch biosynthesis and determines the efficiency of starch synthesis. *MePHD1* can bind to the promoter region of *MeAGPS1a* and act as a negative transcriptional regulator of *MeAGPS1a* expression. Many phytohormones (such as ABA, IAA, and GA) and high temperature (42°C) can upregulate the transcript level of *MePHD1* (Ma et al., 2018). Another study revealed that 10 *PbPHDs* are expressed during pear (*Pyrus bretschneideri*) fruit development. Particularly, the expression of *PbPHD10* showed a similar change pattern to the lignin content with the development of the pear fruit, indicating that *PbPHD10* is a candidate gene for regulating the lignin synthesis (Cao et al., 2018).

### Metal transport

Metal transport from soil to shoots plays an essential role in plant normal growth and is mainly regulated by metal-specific transporters. A PHD finger protein OsTITANIA (OsTTA) is a constitutively expressed transcription factor that can enhance the expression of diverse metal transporter genes in rice. The *tta* mutant, LOW CADMIUM (LC5), displays decreased growth and lower accumulation of several metals, such as zinc (Zn), copper (Cu), and manganese (Mn), in shoots compared to the wild-type plants (Tanaka et al., 2018).

## Roles of PHD finger proteins in response to abiotic stresses

Abiotic stresses are major adverse environment factors limiting plant growth, development, and productivity in the whole world (Wang et al., 2019). During the long-time evolution, plants have formed complicated mechanisms to sense, transmit, and respond to these abiotic stress signals in order to survive and reproduce (Gong et al., 2020). The transcriptional regulation of stress-related genes through transcription factors is an important component of plant stress responses (Wang et al., 2019).

A lot of studies reported that many PHD finger genes are stress responsive and play key roles in plant responses to abiotic stresses, such as salt, drought, and freezing stresses (**Table 2**). Genome-wide *PHD/AL* genes are identified and analyzed in various plant species, including *Arabidopsis* (Zhang et al., 2009; Guk et al., 2022), maize (Wang et al., 2015; Zhou et al., 2017), *B. rapa* (Kayum et al., 2015; Alam et al., 2019), and wheat (*Triticum aestivum* L.) (Pang et al., 2022) (**Table 3**). Wang et al. (2015) reported that 67 PHD finger genes are identified in maize, and 15 *ZmPHDs* are stress-responsive genes detected by promoter *cis*-element and expression analysis. When subjected to PEG, NaCl, and ABA treatments, *ZmPHD14* and *ZmPHD19* are strongly induced or repressed in all stress treatments, while the expression levels of other genes are highly regulated only by one or two treatments. Totally, 73 non-redundant PHD finger genes are isolated from the poplar (*Populus trichocarpa*) genome. Some paralogous genes have high degrees of sequence homology, such as *PtPHD29*/*PtPHD65*, *PtPHD35*/*PtPHD23*, and *PtPHD45*/*PtPHD18*, suggesting that these genes may have redundant functions. In addition, nine genes, for instance *PtPHD68*, *PtPHD31*, and *PtPHD65*, are strongly regulated under drought, salt, or cold stress (Wu et al., 2016b). In moso bamboo (*Phyllostachys edulis*), 60 PHD finger genes are classified into 11 subfamilies according to phylogenetic analysis. Among them, 16 *PePHDs* are stress-responsive genes and differentially induced by drought, low temperature, and NaCl and ABA treatments (Gao et al., 2018). 18 of 145 *BrPHD* genes from *B. rapa* are responsive to drought and salt stresses (Alam et al., 2019). As plant genome annotations are updated, a larger number of previously omitted protein-coding genes are re-identified and re-annotated (Cheng et al., 2017; Kim et al., 2020). Through genome-wide re-annotation of PHD finger genes in tomato (*Solanum lycopersicum*), potato (*Solanum tuberosum*), pepper (*Capsicum annuum*), rice, and *Arabidopsis*, 225 of 875 PHD finger genes were newly identified in the five species, of which 57 is in pepper (Guk et al., 2022). Combining gene expression profiling and GO enrichment analysis showed that many pepper PHD finger differentially expressed genes (DEGs) perform some degree of function in response to salt, heat, or mannitol stress (Guk et al., 2022).

**Table 2:** PHD finger proteins involved in plant response to abiotic stresses.

PHD finger protein	Plant species	Domain	Involved in abiotic stresses	Reference
Alfin1	*Medicago sativa* (alfalfa)	PHD finger	Salt stress	Winicov et al., 2004
GmPHD2, 5, 6	*Glycine max* (soybean)	PHD finger	Salt stress	Wei et al., 2009; Wu et al., 2011; Wei et al., 2017
AtAL7	*Arabidopsis thaliana*	DUF3595, PHD finger	Salt stress	Song et al., 2013
GhCHR	*Gossypium hirsutisms* (cotton)	DC1, PHD finger	Salt stress	Gao et al., 2016
AtAL6	*Arabidopsis thaliana*	DUF3595, PHD finger	Pi deficiency stress	Chandrika et al., 2013
AtAL5	*Arabidopsis thaliana*	DUF3594, PHD finger	Salt, drought and freezing stress	Wei et al., 2015
AhAL1	*Atriplex hortensis*	DUF3595, PHD finger	Salt and drought stress	Tao et al., 2018
MtPHD6	*Medicago truncatula*	PHD finger	Drought stress	Quan et al., 2019
AtSIZ1	*Arabidopsis thaliana*	SAP, PHD finger, PINIT, SP-RING, SXS	Salt and freezing stress, ABA response	Cheong et al., 2009; Miura and Nozawa, 2014; Miura et al., 2020

**Table 3:** Number of *PHD/AL* genes in various plant species.

PHD finger gene	Characteristic domain	Plant species	Number of *PHD/AL* genes	Reference
*AtPHD*	PHD finger	*Arabidopsis thaliana*	70; 257	Zhang et al., 2009; Guk et al., 2022
*GmPHD*	PHD finger	*Glycine max* (soybean)	6	Wei et al., 2009
*MtPHD*	PHD finger	*Medicago truncatula*	7	Wei et al., 2009
*ZmPHD*	PHD finger	*Zea mays* (maize)	67	Wang et al., 2015
*DcPHD*	PHD finger	*Daucus carota* (carrot)	106	Wu et al., 2016a
*PtPHD*	PHD finger	*Populus trichocarpa* (poplar)	73	Wu et al., 2016b
*SlPHD*	PHD finger	*Solanum lycopersicum* (tomato)	45; 92	Chen et al., 2016; Guk et al., 2022
*OsPHD*	PHD finger	*Oryza sativa* (rice)	59; 211	Sun et al., 2017; Guk et al., 2022
*PePHD*	PHD finger	*Phyllostachys edulis* (moso bamboo)	60	Gao et al., 2018
*PbPHD*	PHD finger	*Pyrus bretschneideri* (Chinese pear)	31	Cao et al., 2018
*BrPHD*	PHD finger	*Brassica rapa*	145	Alam et al., 2019
*TaPHD*	PHD finger	*Triticum aestivum* (wheat)	244	Pang et al., 2022
*StPHD*	PHD finger	*Solanum tuberosum* (potato)	209	Guk et al., 2022
*CaPHD*	PHD finger	*Capsicum annuum* (pepper)	106	Guk et al., 2022
*AtAL*	DUF3594, PHD finger	*Arabidopsis thaliana*	7	Wei et al., 2015
*BrAL*	DUF3595, PHD finger	*Brassica rapa*	15	Kayum et al., 2015
*BoAL*	DUF3596, PHD finger	*Brassica oleracea*	12	Kayum et al., 2016
*ZmAL*	DUF3597, PHD finger	*Zea mays* (maize)	18	Zhou et al., 2017
*AhAL*	DUF3598, PHD finger	*Atriplex hortensis*	4	Tao et al., 2018

In cotton (*Gossypium hirsutum*), *GhCHR* encoding a protein with three PHD finger and two DC1 (Cys5-His) motifs can be induced by salt stress and is the target of miRNVL5. Overexpression of *GhCHR* in *Arabidopsis* enhances the tolerance of salt stress with less Na^+^ accumulation in shoots and better primary root growth, compared with the wild type (Gao et al., 2016). The expression of an *M. truncatula* gene, *MtPHD6*, can be induced by osmotic and drought stresses. *MtPHD6*-overexpressing *Arabidopsis* plants display lower MDA and ROS contents and higher leaf water content and antioxidant enzyme activities than the wild-type plants under drought stress, leading to the enhanced drought tolerance. Moreover, they found that the transformation of *MtPHD6* predominantly upregulates the expression of *WRKY*, *ZINC FINGER*, and *AP2/EREBP* transcription factors (Quan et al., 2019). Plant SIZ proteins encode SUMO (small ubiquitin-related modifier) E3 ligases that play key roles in sumoylation (Jin et al., 2008). AtSIZ1 contains a plant-specific PHD finger domain, while the orthologs in yeast and animals have no PHD finger. The mutation in the PHD finger domain impairs the SUMO conjugate formation and generates the long-hypocotyl phenotype related to sugar and light conditions (Cheong et al., 2009). The PHD finger of AtSIZ1 recognizes H3K4me3, which is important for the suppression of *WRKY70* expression and for the interaction with ATX1, a histone lysine methyltransferases. AtSIZ1 without the PHD finger or with mutated PHD finger does not complement the freezing sensitivity and drought tolerance induced by the *siz1-2* mutant, whereas AtSIZ1 does, indicating that the PHD finger is necessary for AtSIZ1 function as a transcriptional repressor (Miura et al., 2020). Additionally, the overexpression of *AtSIZ1* enhances the tolerance of transgenic plants to freezing and salinity stresses and reduces the inhibition induced by ABA treatment (Miura and Nozawa, 2014).

As a plant-specific subfamily of the PHD finger proteins, ALs normally possess transcriptional suppression activity and play crucial roles in biological processes by inhibiting expression of downstream target genes (Wei et al., 2017). Most *AL* genes are also significantly induced by abiotic stresses, such as cold, salt, drought, and ABA treatment (Wei et al., 2009; Wei et al., 2015; Tao et al., 2018). Of the 12 *AL* genes from *B. oleracea*, 10 are abiotic stress responsive (Kayum et al., 2016). In soybean, Wei et al. (2009) identified six Alfin1-type PHD finger proteins, GmPHD1-6, with the ability of binding to the cis-element “GTGGAG.” GmPHDs have transcriptional suppression activity except GmPHD6. Heterologous expression of *GmPHD2* in *Arabidopsis* inhibits the expression of seven negative regulator genes, such as *DREB1C*, *STRS1*, and *STRS2*. Eight genes are upregulated in transgenic plants, including *ABI5*, *WAK5*, *GLP*, *MDAR*, *TPP*, and three peroxidase genes. Expression changes in these stress-responsive genes showed that *GmPHD2* enhances salt tolerance though affecting stress signaling and by eliminating ROS in transgenic plants (Wei et al., 2009). Under salt stress, GmPHD5 regulates the crosstalk between the methylated H3K4 and the acetylated H3K14 and may recruit chromatin remodeling factors and transcription factors to modulate the transcription of stress-inducible genes, including *GmRD22* and *GmGST* (Wu et al., 2011). *GmPHD6* overexpression improves the tolerance to salt stress in soybean through interacting with LHP1 by the PHD finger domain. The GmPHD6 and LHP1 form a transcriptional activation complex to activate expression of downstream stress responsive genes, such as *CYP82C4*, *CYP75B1*, and *CCD7*, suggesting that the GmPHD6-LHP1 complex plays a key role in salt tolerance (Wei et al., 2017). In *Arabidopsis*, seven *AL* genes are identified and functionally characterized. *AtAL6* is essential for the formation of root hair during phosphate (Pi) deficiency stress. It binds to H3K4me marks at the Myb-type transcription factor *ETC1* through its PHD finger domain, which may facilitate the transcription of downstream Pi-responsive genes (Chandrika et al., 2013). The T-DNA insertion mutants of *AtAL7* display enhanced salt tolerance with longer root length, indicating that *AtAL7* functions as a negative regulator in response to salt stress (Song et al., 2013). Overexpression of *AtAL5* improves the tolerance of transgenic plants to salt, drought, and freezing stresses by repressing the transcription of downstream negative regulatory genes, including *SHMT7*, *TAC1*, *OFE*, *FAO*, and *CAX1* (Wei et al., 2015). In addition, four *AhAL* genes are isolated from *A. hortensis* and encode nuclear-localized proteins with the transcription repression activities. *AhAL1*-transgenic *Arabidopsis* shows the higher survival rate under salt and drought stresses by reducing MDA content and water loss. Through binding to the promoter regions of target genes, *AhAL1* represses the expression of negative regulator genes in ABA signaling, such as *DREB1C*, *GRF7*, and five group-A protein phosphatase 2C (PP2C)-encoding genes (*ABI1*, *ABI2*, *AHG3*, *HAB1*, and *HAB2*), which then induces the activation of some ABA/stress-responsive genes, including *DREB1A*, *DREB2A*, and three genes encoding ABA-responsive element (ABRE)-binding factors (*ABF*2, *ABF3*, and *ABF4*) (Tao et al., 2018). In conclusion, the overexpression of PHD finger genes can improve abiotic stress tolerance of transgenic plants with better growth phenotype. PHD finger genes exert the transcriptional regulatory activity by inhibiting or activating the expression of downstream stress-related genes in plant adaptation to adverse environment.

## Conclusion and perspectives

**Figure 1:**
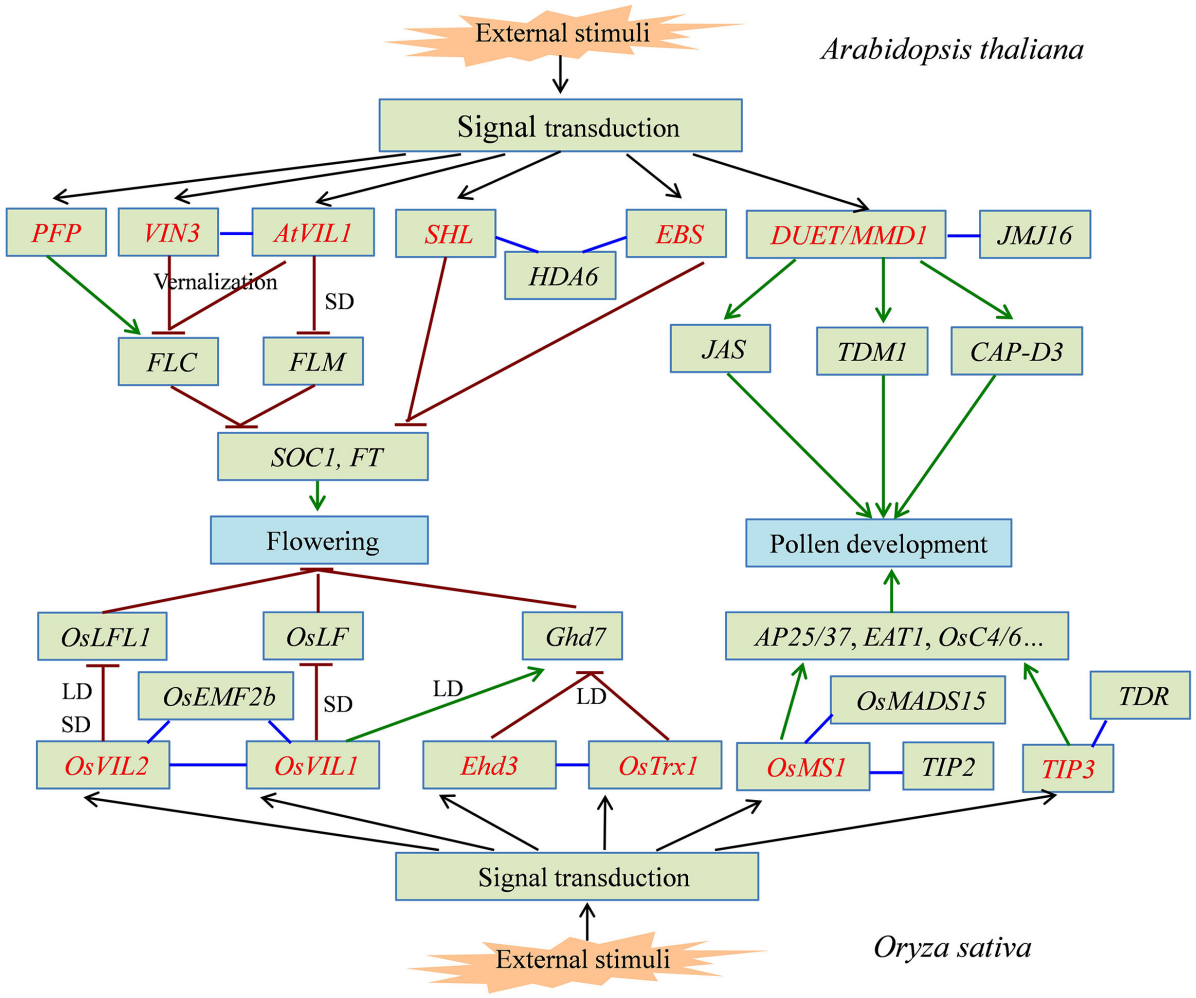
The signal pathway of partial PHD finger proteins involved in plant flowering and pollen development. PHD finger proteins from *Arabidopsis thaliana* and *Oryza sativa* directly or indirectly activate (green line) or inhibit (dark red line) the expression of downstream target genes, which can precisely regulate the flowering time and normal pollen development in plants. PHD finger proteins are shown in red font. The interaction between two proteins is shown in blue line.

**Figure 2:**
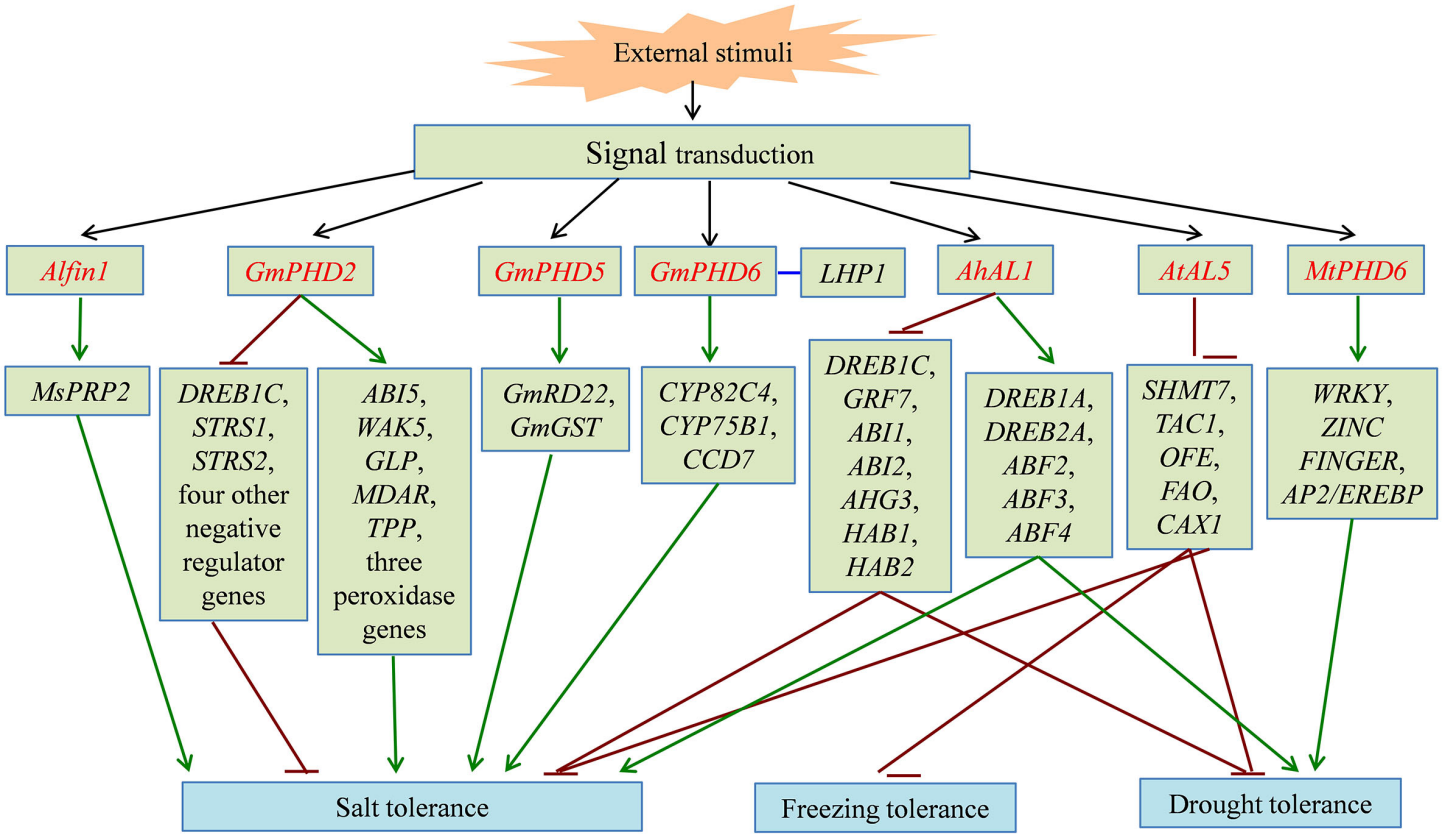
The signal pathway of partial PHD finger proteins involved in plant abiotic stress responses. PHD finger proteins directly or indirectly activate (green line) or inhibit (dark red line) the expression of downstream stress-related genes, and enhance tolerance to salt, freezing, and drought stress in different plant species. PHD finger proteins are shown in red font. The interaction between two proteins is shown in blue line.

**Figure 3:**
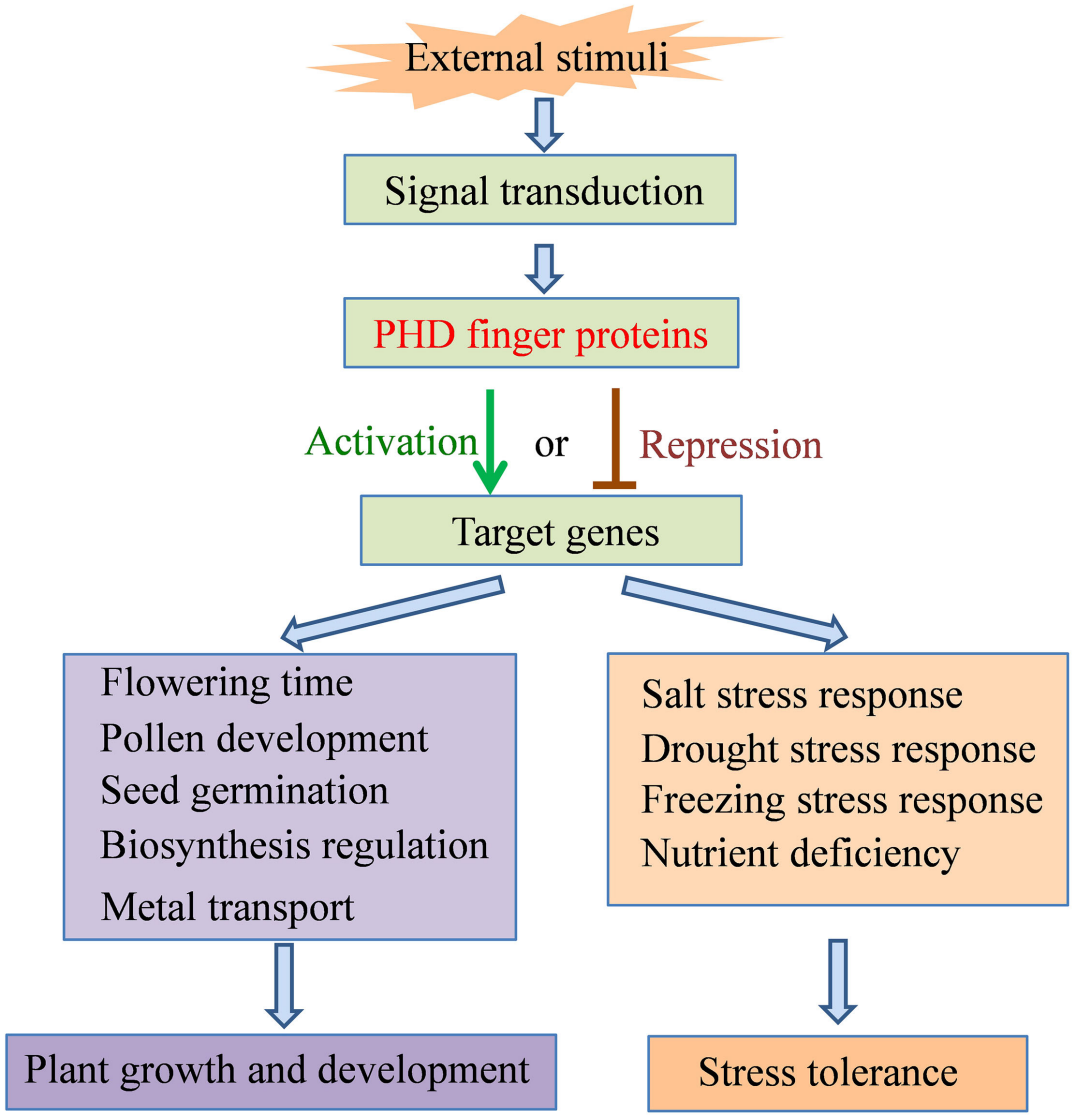
Summary framework of the roles of PHD finger proteins in plant growth and development and stress tolerance.

Proteins containing the PHD finger domain are widespread in plants and are one of the important transcription regulator families. Recently, many PHD finger proteins are identified in diverse plant species and proved to be involved in various biological processes. In this review, we mainly focused on the roles of PHD finger proteins in plant reproduction development, such as floral transition, tapetum development, and male meiosis (**Figure 1**), and responses to abiotic stresses, including salinity, drought stress, and low temperature (**Figure 2**). Moreover, acting as key transcriptional regulators, some PHD finger proteins also function in metal transport and biosynthesis pathways, which are important for the normal growth of plants. In summary, after being exposed to external stimuli, PHD finger proteins can bind to specific regions of downstream target gene promoters through the PHD finger to exert its transcriptional regulatory activity, activate or inhibit the expression of responsive genes related to plant growth and development and stress response, and finally achieve the role of regulating plant development and stress tolerance (**Figure 3**).

The PHD finger is the “reader” domain of epigenetic modification and directly binds to the methylated histone H3, which plays key roles in chromatin remodeling and transcription regulation of target genes (Sung and Amasino, 2004; Wu et al., 2011; López-González et al., 2014; Wang et al., 2016; Miura et al., 2020). Furthermore, PHD finger proteins can interact with other proteins through the PHD finger domain to regulate specific biological processes. For example, the interaction of OsMS1 with TIP2 and OsMADS15 is necessary for the tapetum development and pollen wall formation, both of which play crucial roles in the production pf mature pollen grains (Yang et al., 2019a). In order to verify the functional importance of the PHD finger, the target gene with or without the PHD finger domain is transferred into the corresponding mutant plants to observe whether the abnormal phenotypes can be restored. For instance, under the control of the *AtSIZ1* promoter, the recombinant vector containing *AtSIZ1* gene with or without the PHD finger is transferred to the *siz1-2* mutant. The expression of *Pro_SIZ1_::SIZ1:GFP* could be able to complement the defective phenotypes of the *siz1-2* mutant, while *Pro_SIZ1_::SIZ1(ΔPHD):GFP* does not, demonstrating that the PHD finger is critical for AtSIZ1 in plant response to cold stress, drought stress, and ABA treatment (Miura et al., 2020).

With the development of transcriptome-wide sequencing and the updating of genome-wide annotation, more and more PHD finger genes will be excavated or be re-annotated in plant species; however, further studies still need to focus on the exact functions of PHD finger proteins. For instance, many PHD finger genes are proved to be stress responsive, but their biological functions in plant responses to abiotic stresses remain to be confirmed. By using PHD finger gene-specific overexpression and mutant lines is helpful for analyzing their functions. In addition, it is worth noting that a PHD finger protein may have multiple roles participated in different biological events, such as AtAL6 (Chandrika et al., 2013; Molitor et al., 2014) and AtSIZ1 (Jin et al., 2008; Miura et al., 2020). Last but not the least, the signaling transduction pathways by which many PHD finger proteins perform specific biological functions are still unclear. At present, the research on some PHD finger proteins is limited to the identification of biological functions. The molecular mechanism of its specific biological function has not been studied deeply. Many studies have screened differentially expressed genes through transcriptome sequencing, so as to obtain downstream functional genes modulated by a PHD finger protein. The upstream regulators of this protein expression and their functions have been less studied. Identifying the upstream and downstream interaction factors of PHD finger proteins is essential to better understand their molecular mechanisms in a biological process. The yeast two-hybrid assay and other protein interaction methods are useful for analyzing and verifying proteins interacted with PHD finger proteins. To sum up, the in-depth research on the biological functions of PHD finger proteins and the construction of their molecular regulatory networks will enrich and improve our understanding of the roles of PHD finger proteins in various biological events. Meanwhile, this will also provide a valuable scientific basis for the study of new PHD finger proteins. In addition, the PHD finger protein encoding genes may be used as novel candidate genes to modify the plant genome for enhancing the tolerance of transgenic plants to adverse environment or improving their growth and development, ultimately leading to the plant biomass increase.

## Author contributions

WQ: Writing – original draft. ZC: Writing – review & editing. PW: Writing – review & editing. YM: Writing – review & editing. DB: Writing – review & editing. XL: Writing – original draft.
